# “I feel like I’m walking on eggshells”: a qualitative study of moral distress among Chinese emergency doctors

**DOI:** 10.1186/s12910-024-01074-4

**Published:** 2024-06-20

**Authors:** Jiajun Liu, Fengling Dai, Qitai Song, Jian Sun, Yao Liu

**Affiliations:** 1 School of Nursing, Southwest Medical University, Luzhou, Sichuan Province 646000 China; 2 Department of Emergency Medicine, The Affiliated Hospital, Southwest Medical University, Luzhou, Sichuan Province 646000 China

**Keywords:** Emergency Department, Moral Distress, Qualitative research, Doctors

## Abstract

**Background:**

While the number of emergency patients worldwide continues to increase, emergency doctors often face moral distress. It hampers the overall efficiency of the emergency department, even leading to a reduction in human resources.

**Aim:**

This study explored the experience of moral distress among emergency department doctors and analyzed the causes of its occurrence and the strategies for addressing it.

**Method:**

Purposive and snowball sampling strategies were used in this study. Data were collected through in-depth, semi-structured interviews with 10 doctors working in the emergency department of a tertiary general hospital in southwest China. The interview data underwent processing using the Nvivo 14 software. The data analysis was guided by Colaizzi’s phenomenological analysis method.

**Study findings:**

This study yielded five themes: (1) imbalance between Limited Medical Resources and High-Quality Treatment Needs; (2) Ineffective Communication with Patients; (3) Rescuing Patients With no prospect of treatment; (4) Challenges in Sustaining Optimal Treatment Measures; and (5) Strategies for Addressing Moral Distress.

**Conclusion:**

The moral distress faced by emergency doctors stems from various aspects. Clinical management and policymakers can alleviate this distress by enhancing the dissemination of emergency medical knowledge to the general public, improving the social and economic support systems, and strengthening multidisciplinary collaboration and doctors’ communication skills.

**Supplementary Information:**

The online version contains supplementary material available at 10.1186/s12910-024-01074-4.

## Background

Emergency medicine is an essential worldwide discipline that aims to avert secondary illnesses and functions as a vital instrument for implementing basic disease prevention [1]. Emergency departments (ED) play a crucial role in healthcare worldwide, offering essential, continuous, and inclusive healthcare services to all patients [2]. In contemporary healthcare facilities, the ED has constantly served as a central point for admitting patients to the hospital [3]. Due to the rising number of visitors to the ED worldwide [4], overcrowding in the ED has emerged as a prevalent issue in healthcare systems across the globe [5]. Due to the urgent and critical condition of most patients in the ED, healthcare personnel are often required to make prompt and precise medical decisions. These decisions encompass matters of life and death, allocation of medical resources, patient dignity, and family preferences. Research indicates that timely and appropriate on-scene decision-making can lead to significantly positive outcomes. However, medical decision-making is a complex and ever-changing process influenced by cultural context, interactions, personal feelings, and a multitude of factors [6]. Consequently, healthcare personnel in the ED are susceptible to experiencing significant moral distress in this setting.

The term “moral distress” was initially used by Andrew Jameton in 1984. It describes a form of ethical anguish experienced when an individual is aware of the need to act ethically but is unable to do so due to internal or external constraints. This phenomenon falls under the broader category of ethical conflict [7, 8]. Moral distress arises from a conflict or worry related to ethical matters [9], while moral distress is fuelled by ethical challenges [10, 11]. The causes of moral distress can be ascribed to the doctor-patient conflict arising from patients’ insufficient understanding of emergency treatment, the incongruity between the demands of emergency care and available human resources [12], and the disparities in healthcare resources among regions [13]. Prolonged moral distress can have various detrimental effects on healthcare workers. These effects may include feelings of threat, powerlessness, guilt, or confusion [14]. Furthermore, they can lead to occupational burnout, empathy fatigue [15, 16], increased turnover [17, 18], diminished quality of care [15, 19], and even hypervigilance or distraction, which can subsequently result in adverse events [20, 21].

Chinese emergency medicine doctors are also experiencing noticeable moral distress, adding to the shortage of personnel [22]. The substantial population size and growing elderly population in China have resulted in considerable strain on healthcare practitioners [23]. In 2017, the annual ED admissions in China surpassed 166.5 million [24]. Due to the impact of Chinese Confucian culture, family members hold significant power in medical practice [25, 26]. Consequently, clinicians must exert substantial effort in communicating effectively with family members when confronted with intricate medical issues. Of Chinese emergency doctors, precisely 78.39%, experience a crucial imbalance in their give-and-take interactions [23]. Additionally, 55.18% of Chinese emergency doctors have a propensity to resign from their positions [12]. The high rate of turnover among emergency doctors is a significant difficulty for the Chinese healthcare system [27].

Researchers worldwide have been concentrating on matters pertaining to moral distress among healthcare providers in ED. Emergency doctors in Pakistan have reported encountering regular obstacles in their everyday work. They believe that ethical principles are at risk and that ethical decision-making is necessary alongside clinical decision-making [28]. Bruun et al. employed a practice-based analytic model to demonstrate the emergence of ethical challenges in the context of pre-hospital emergency care, as well as in the course of providing care and engaging in external partnerships [29]. Foster et al. discovered that moral distress can arise when the best care for patients cannot be provided [30]. Researchers in Stockholm have proposed that the exhaustion of healthcare resources is a frequent reason for moral distress experienced by ED doctors [31]. According to Clark et al., there is a correlation between reduced job satisfaction among ED nurses and greater scores of moral distress [32]. Nevertheless, there has been a scarcity of research that examines explicitly the moral distress experienced by ED doctors in China, and there has been a dearth of comprehensive exploration into their profound personal encounters. Understanding the moral distress experienced by emergency doctors can provide a reference for proposing effective improvement measures.

### Study aim

This study aimed to explore the moral distress faced by doctors working in EDs in clinical practice and to provide a reference for alleviating the moral distress experienced by emergency doctors.

## Method

### Study design

The philosophical underpinnings of Husserlian phenomenology are those of the lived human experience. The rich and complex source of unspoken meaning associated with being and experiencing shapes an individual’s understanding of their life-world. Elucidating and examining the process of forming key concepts by retracing the original experience of consciousness can gain unequivocal proof for the intended actuality of these concepts [33]. Consequently, conducting analyses of conscious experience should be approached from the perspectives of those encountering it directly [34]. Our descriptive phenomenological study was conducted based on Colaizzi’s method of analysis [35], which attempts to capture the raw essence of the moral distress experienced by emergency doctors from their point of view.

This study, involving male and female researchers, was conducted by a multidisciplinary team comprising two senior ED MMed doctors, a nursing PhD, and two nursing students. The team utilized diverse expertise: the doctors contributed invaluable clinical insights and firsthand experiences with ethical challenges; the nursing PhD provided a robust theoretical and academic foundation; and the nursing students actively engaged in practice, offering fresh perspectives. All team members were thoroughly trained in qualitative research methodologies, ensuring the study’s methodological rigor.

This study was designed and reported following the consolidated criteria for reporting qualitative studies (COREQ) checklist (see “Additional File 1”) [36].

### Study setting

This study was conducted in a general tertiary hospital directly under the Sichuan Provincial Health Commission, a top-tier comprehensive hospital. In China, tertiary hospitals represent the highest tier in a three-level classification system that ranks hospitals based on their capabilities in medical care, education, and research. These institutions often function as regional centers for comprehensive and specialized medical services [37]. Founded in 1950, it serves nearly 60 million people in Sichuan, Chongqing, Yunnan, and Guizhou provinces [38]. The ED of this hospital is a nationally recognized advanced stroke prevention and treatment center, chest pain center, and maternal and child emergency care unit. It handles over 140,000 patient visits annually, averaging 328 visits per day. The department performs over 7,000 emergency rescues yearly, with a success rate exceeding 97%. Additionally, it provides on-site medical treatment for more than 3,000 patients annually [39]. We chose this hospital due to its extensive and diverse patient base, providing a comprehensive medical practice perspective.

### Participants

Purposive and snowball sampling was used to recruit informants from targeted hospital. Doctors employed in the ED for more than six months were chosen. The interviewer and participants were not acquainted prior to the interviews. The sample size was reached when data saturation occurred, and no new information was generated.

Data saturation is a phenomenon in qualitative research that occurs when further interviewing ceases to yield novel concepts and themes [40]. In contrast to quantitative research, which necessitates a larger sample size for support, qualitative research favors sample selection that is more targeted. This study employed a total of ten interviews plus an additional interview to reach the data saturation, which is within the acceptable range for qualitative research saturation, which is between 9 and 17 interviews [41]. Researchers (JL, FD, YL) conducted detailed meetings and saturation analyses of themes and codes after each interview to determine data saturation. When no new information emerged from consecutive interviews, we considered data saturation to be reached [42]. This approach ensured the completeness and depth of our research data while avoiding the collection of redundant information.

### Data collection

To objectively describe the participants’ experiences, we conducted interviews and data analysis using a multidisciplinary team approach, possible to set aside personal preconceived notions about the phenomena [43]. Data were collected by the principal investigators (JL, YL, QS). Prior to the interviews, the participants were provided with comprehensive information regarding the study’s objectives, approach, and procedure by YL and QS. To guarantee the confidentiality of the participants and enable them to articulate their perspectives openly, privacy, comfort, and convenience were considered in selecting the interview location. Consent was obtained from the interviewees, and the audio recording was used after getting the permission of the participants. Participants had opportunities to ask questions, refuse questions, and withdraw from study participation at any time. The participants were motivated to recall instances of cases from their clinical experiences in which they elaborated on the specific moral distress that arose and the manner in which they resolved them. Interviews were conducted in Mandarin Chinese by author JL. Interview times ranged from 45 to 70 min, and interviews were conducted until no new information was forthcoming. No participants withdrew from the study. Facial expressions and body language were captured through handwritten notes.

All authors collaborated to design an interview guide aimed at investigating ED doctors’ perceptions of moral distress. A preliminary interview guide was generated from a literature review, and it was refined through expert feedback. Two cases of pre-interviews were conducted to check the rationality of the interview guide and the feasibility of the interview.

The interview process begins with general questions, such as “Could you provide examples of moral distress you commonly encounter in the ED?” and “What is the frequency of experiencing such events?” to establish a basic conceptual framework. Once the foundational concepts are established, probing questions, such as “Could you describe these events specifically?” and “What kind of emotions do they evoke in you?” are refined based on gaps identified in previous interviews and emerging themes [6]. Consequently, the questions in different interviews are tailored to the specific context of each interview to ensure a thorough exploration of each participant’s unique experiences and perspectives. The finalized interview guide provides specific examples of interview questions (see “Additional file 2”).

### Analysis

Colaizzi’s method was used to guide the data analysis [35]. It was divided into seven analytical steps: The researcher’s collective methodology was used to gain familiarity with the research data, identify meaningful statements, and construct codes (JL, FD, JS).

(1) The documentation was compiled and transcribed word for word in Microsoft Word within a 24-hour timeframe following the interviews by authors JL and JS [44]. Recorded interviews were transcribed verbatim in Mandarin. The interview transcription form was used to record non-verbal behaviors, resulting in a final transcribed document containing 140,712 words.

(2) The two researchers (JL, FD) respectively and repeatedly read the interview transcripts to immerse themselves in the data. Two cases of transcripts were returned to the participants to verify. Data were uploaded into NVivo 14 for coding and management. As data collection progressed, data were continuously added to NVivo, and analysis was conducted iteratively to ensure comprehensive coding.

(3) Identify important statements related to the moral distress of ED doctors.

(4) Extract meaningful excerpts, and conduct coding to identify commonalities and differences(JL, FD). Through team discussions (JL, FD, JS) and cross-checking by co-authors. iteratively revise the analysis until the research team reaches a consensus, this minimized errors and enhanced credibility and confirmability [45].

(5) Organize each important statement into meaningful units and sub-themes, then consolidate them into main themes.

(6) Closely link and describe the themes in detail with the research phenomenon.

(7) Feedback results to participants (P01, P07) to further validate the credibility and validity of the findings.

In addition, to ensure semantic accuracy, two bilinguals scrutinize the raw data and themes.

### Findings

The participants were evenly distributed between males and females, with a mean age of 36 years. Out of the total, the majority (9/10) possessed a master’s degree or higher. In terms of occupation, both physicians and surgeons were equally represented. Additionally, the majority (8/10) had at least 5 years of experience in the ED. The characteristics of participants are seen in Table 1.

**Table 1:** Participant characteristics

Attribute	No. of participants *N* = 10	Male	Female
ED position			
Physician	5	1	4
Surgeon	5	4	1
Highest level of education attained			
Bachelors	1	0	1
Masters	7	4	3
MD	2	1	1
Duration of work in ED			
6 months-1 year	2	0	2
1–5 years	1	1	0
6–10 years	3	3	0
11–15 years	3	1	2
16–20 years	1	0	1
Titles for Medical Doctors			
Junior doctors	6	3	3
Intermediate doctors	3	2	1
Senior doctors	1	0	1
Age			
20–29	2	0	2
30–39	4	3	1
40–49	4	2	2

The data analysis yielded five main themes, see Table 2.

**Table 2:** The Moral Distress and Strategies for Addressing Moral Distress of Emergency Doctors

Main themes	Subthemes
Imbalance between Limited Medical Resources and the High-Quality Treatment Needs	▸ Stringent Time Constraints in Diagnoses ▸ Inadequate Human Resources in Emergency Departments ▸ Overcrowding of Emergency Medical Resources by Non-emergency Patients
Ineffective Communication with Patients	▸ Sudden and critical illness exacerbates anxiety among family members ▸ Distrust of Healthcare Personnel by Patients and Their Families
Rescuing Patients With no prospect of treatment	▸ Emotional Requests from Family Members ▸ Economic Considerations Driving Family Members’ Decisions
Challenges in Sustaining Optimal Treatment Measures	▸ Lack of Sustained Financial Support for the Patient ▸ Lack of Support for Therapeutic Decision-Making ▸ Lack of Sufficient Multidisciplinary Collaboration ▸ Elevated Levels of Liability Risk
Strategies for Addressing Moral Distress	▸ Enhancing the Dissemination of Emergency Medical Knowledge to the General Public ▸ Refining the Social and Economic Support Systems ▸ Strengthening Multidisciplinary Collaboration ▸ Improving Doctors’ Communication Skills

#### Imbalance between Limited Medical Resources and the high-quality treatment needs

The majority of interviewees (P03, P04, P06, P07, P09, P10) stated that the short turnaround time for diagnosis in ED, the general absence of human resources for medical care in EDs, and the excessive use of emergency medical resources by non-emergency patients were the leading causes of the disparity between the supply of emergency medical resources and the demand for high-quality care for ED patients.

#### Stringent time constraints in diagnoses

The ED where this study was conducted averages 328 patients per day. The intricate nature of the condition in the ED setting presents challenges for ED doctors to exclude other systemic disorders and establish a meticulous diagnosis within the constrained timeframe.

A junior internal medicine physician with 13 years of experience pointed out that the time to handle each patient is extremely limited, and the assessments are rushed. *“The typical period from when they arrive at our ED to when they leave is probably approximately a half hour to an hour at most. If the patient has an upper respiratory illness, the time would be even shorter. "* (Participant 07 [P07])

A young internal medicine physician who had just started their career six months ago further explained: *“Our diagnosis time is also limited; we need to assess the patient’s condition and carry out emergency treatment within a very limited time. Sometimes, if the patient himself did not recall certain symptoms or medical history at that time, and the examination also did not reflect any changes in the condition, the patient would go home, but his situation suddenly deteriorated after returning home.”* (P04) Time constraints may lead to the risk of missing critical information, resulting in severe consequences after the patient is discharged.

#### Inadequate Human resources in Emergency Departments

It is challenging for ED doctors to constantly monitor the altering symptoms or emotions of each patient due to the shortage of healthcare personnel in the ED.

An intermediate internal medicine physician with 13 years of experience described this situation: *“It is true that we are now experiencing a severe human resource deficit in our unit. Two or three nurses on duty at night oversee more than ten or twenty patients. In addition to the ward, there are ten or twenty patients in our emergency room and observation room. It takes one minute for each patient to spend a lot of time, let alone solve the problem in just one minute.”* (P06).

A junior Surgeon with six months of experience added: *“The fundamental distress of emergency work lies in the large number of patients and insufficient personnel, including nursing staff. Each healthcare worker must deal with multiple patients at the same time… For example, patients will complain that their fluids have run out and that no one has come over to take their syringes in a timely manner. With a low doctor or nurse-to-patient ratio, there is no way to avoid this problem completely; unlike outpatient clinics, where patients can be seen one after another, we should deal with many patients at the same time, and it’s hard to keep an eye on each of them at all times.”* (P03).

These situations highlight the significant impact of staff shortages on the ability of healthcare personnel to provide continuous monitoring and care.

#### Overcrowding of Emergency Medical resources by Non-emergency patients

Due to the lack of clarity surrounding the ED’s scope of care, the majority of interviewees indicated that non-emergency patients utilized the ED’s expediency to present themselves there.

*“There are many non-emergency patients in the ED. Some patients say they have to work during the day and do not have time to visit the outpatient clinic; they can only see the doctor at night. Some patients believe that the emergency clinic needs to deal with patients on time so the examination report will be out faster, and they will not have to queue up. Many people would exploit this loophole (helpless).”* (P03).

This reflects the broader issue of convenience and perceived efficiency driving patients to misuse emergency services.

Additionally, the lack of proper triage for the influx of non-emergency patients into the ED contributes to a chaotic and boisterous environment. It is also prone to conflicts.

*“There is a lack of triage for many mild patients. in a situation where they may perceive their condition as urgent. Consequently, both mild and severe cases flood the ED simultaneously. In situations of scarce medical resources, doctors prioritize rescuing severe cases. However, patients without urgent indications may believe they should be treated first because they get here earlier, leading to conflicts when others intervene and demand treatment. This dynamic creates tensions between healthcare providers and patients.”* (P04).

During large-scale mass casualty incidents, such as natural disasters, the number of patients often exceeds the emergency department’s capacity, straining medical resources to their limits. In these scenarios, triage becomes essential to prioritize those with the most critical needs. However, this necessary focus on severe cases can leave less critical patients feeling neglected, escalating tensions and conflicts within the already stressed environment. Such situations not only challenge the efficiency of emergency response but also test the resilience and communication skills of healthcare providers.

### Ineffective communication with patients

The diversity of the patient population at the research site further complicates communication dynamics. Due to anxiety caused by severe illnesses or the lack of faith in healthcare professionals by patients and their families, communication barriers between doctors and patients are often hindered.

#### Sudden and critical illness exacerbates anxiety among family members

In real-life situations, family members are often not emotionally prepared for the patient’s severe sickness, and the overwhelming shock and sadness leave them in a state of worry that hinders their ability to have a calm discussion with the doctor regarding additional medical choices.

As noted by a junior surgical doctor with 8 years of experience: *“Unlike chronic illness with a gradual adaptation period, acute incidents, such as a car accident, are mentally challenging. Patients who were lively and optimistic one day could become critically ill after a sudden accident. Communicating treatment plans with the patient’s family at this juncture becomes a complicated and challenging task.”* (P01).

*“Communication with ED patients and their families is more difficult than in other departments, such as the inpatient department. This is because there are more severely sick patients in ED, and family members who are in certain emotional states fail to take medical advice seriously.”* (P03).

#### Distrust of healthcare personnel by patients and their families

Additionally, several interviewers noted that certain patients or family members believe that doctors pursue their personal interests by excessively prescribing costly medical treatments.

A junior Surgeon with 5 years of experience explained, *“The family members distrust the medical professionals, and they believe that they are being tricked out of money by having the test done or that hospitalization is unnecessary. In this situation, communication is difficult. For some family members, he would ask repeatedly, and even if you point out to him that a decision for the patient is the best, he may still not listen. He may even wonder whether you are scheming anything else for him.”* (P02).

The ultimate victims of such mistrust are the patients, as treatment delays may lead to a loss of their optimal therapeutic opportunities:

*“Take a heart attack, for instance. We explain to them that it’s a serious matter, urgent treatment is crucial, and now is the best time for treatment. However, they perceive it as something they can tolerate, thinking it hasn’t affected their current lifestyle significantly, and thus, they don’t prioritize it.”* (P01).

Some interviewees explicitly stated that the fundamental reason for the lack of trust in healthcare professionals might be the adverse media reports aimed at garnering attention. In situations where the “media fails to understand the facts” (P09), they engage in detrimental reporting practices, often distorting the narratives about healthcare professionals by “taking things out of context” (P08). This adverse reporting is highlighted in various instances (P01, P03, P08, P09, and P10). “It has significantly impacted the entire emergency care environment” (P03, P09):

An experienced internal medicine physician emphasized, *“The media have no understanding of the real situation and are completely talking nonsense, and there is no organization to monitor them. They believe that the story is written to garner a lot of attention; they only consider the amount of airplay and retweets; they do not consider the impact and consequences on a society’s overall medical aspect, and these media outlets are the primary cause of the doctor-patient conflict’s escalation.”* (P09).

### Rescuing patients with no prospect of treatment

Most interviewees (P01, P02, P03, P05, P07, P10) highlighted that families persist in requesting resuscitation and treatment for patients with no prospect of treatment. This might be attributed to the *“emotional reluctance of the family”* (P03) or the desire to provide an opportunity for “ *other family members to have a final encounter with the patient* ” (P02, P05). Certain relatives may insist on prolonged patient treatment without any therapeutic benefit, *“ motivated by financial gain ”*(P02, P07, P10). The ED of this hospital handles over 140,000 patient visits annually, making it a key setting for understanding these dynamics. These situations underscore the complex interplay of emotional and economic motivations in medical decision-making, highlighting the need for better communication strategies and ethical considerations in patient care.

#### Emotional requests from family members

Some informants said that many patient’s family members are emotionally incapable of accepting the patient’s illness as it is and demand that the patient continue receiving treatment.

*“Some family members are mentally unable to accept the (progress of the disease) and insist on continuing to rescue the patient, even if the patient’s vital signs have entirely vanished and resuscitation has been going on for half an hour or more. This has lost the meaning of resuscitation, and continuing the resuscitation means that the patient’s body will continue to inflict injury since it will continue to give him intravenous fluid perfusion and then chest compressions, which affect the remains.”* (P03) These emotional requests often stem from deep-seated denial and hope, as families struggle to accept the impending loss of a loved one. The cultural context also plays a role, where continuing treatment is seen as a way to show filial piety and respect (P02, P03).

#### Economic considerations driving family members’ decisions

A number of interviewees perceived that lengthening a patient’s life could lead to financial benefits for their family members. This, in turn, may lead to requests for extended treatment and resuscitation.

An intermediate Surgeon with 10 years of experience expressed their thoughts: *“From my perspective, it seems like the family insists on keeping the patient alive even when there’s no real hope for treatment. They opt to prolong hospital stays, especially if the patient’s insurance coverage is substantial and treatment costs are minimal. With a steady retirement income, the family is more inclined to continue treatment. However, these interventions sustain the patient’s life without offering any chance of true recovery. …Sadly, in such situations, the patient’s dignity is often neglected.”* (P10).

This perspective highlights the intersection of economic and ethical considerations in medical decision-making. In some cases, financial incentives may overshadow the patient’s best interests, leading to prolonged suffering and loss of dignity.

### Challenges in sustaining optimal treatment measures

The factors that most interviewees (P01, P02, P03, P05, P06, P07, P08) find distress include a lack of financial support for treatment, insufficient support in treatment decision-making, inadequate multidisciplinary collaboration, and the high personal liability risk faced by doctors. These challenges make it difficult for patients to sustain treatment and for doctors to implement optimal treatment measures.

#### Lack of sustained financial support for the patient

Although some patients clearly have certain treatment prospects, the lack of sustained economic support may lead patients or their families to discontinue treatment:

A senior internal medicine physician with over 20 years of experience stated: *“We believe these patients have an excellent prognosis, and we try our best to provide them with treatment. However, due to the patient’s economic situation, they may tell you that they cannot afford the treatment, and they choose to give up. Actually, for us doctors, we are very upset.”* (P05).

According to many informants, the decision-making power of patient treatment sometimes lies with the family members. Patients and families may choose to discontinue treatment due to the apprehension that “even if they spend money on treatment, they may not see positive results” (P08) or because they “wish to avoid placing a burden on their children and families.” (P02).

#### Lack of support for therapeutic decision-making

When a patient is in a critical condition and needs a family member to choose and approve a treatment plan promptly, the family member cannot make decisions on time due to concerns about potential responsibility.

*(Dependents) They’re afraid of taking responsibility and refuse to make any decisions. Our rescue efforts are limited to maintaining basic vital signs, which could delay treatment, especially in emergencies like cerebral hemorrhage. We try to prepare for surgery within half an hour and immediately get the patient into the operating room. However, the dependents can’t seem to decide to approve the medical options. (P03)*

This hesitation and fear of responsibility can significantly impact the timeliness and effectiveness of emergency interventions.

#### Lack of sufficient multidisciplinary collaboration

Half of the interviewees (P01, P02, P03, P05, P07, P09) indicated that emergency conditions often require interdisciplinary collaboration for treatment. However, there are some time instances of poor collaboration between departments, resulting in inefficient patient turnover:

*“Indeed, the turnover rate for patients is often quite slow, and one issue lies in how the inpatient department manages patient placement; we don’t have an opportunity to understand it clearly. Sometimes, we even encounter situations where no emergency patient is admitted for an entire day, which is genuinely problematic. … Moreover, the way bed allocation is handled there seems opaque. ”*(P09).

Some departments may have differences of opinion regarding admission criteria:

*“Well, in reality, everyone prefers to treat patients who are easy to manage and who are more compliant. It’s possible that if you consult five different departments, they might not entirely agree on which department’s disease it is. For instance, a patient with a respiratory condition might also have concurrent heart or kidney issues. The respiratory department might think it’s a respiratory difficulty due to heart problems, while the cardiology department might believe it’s respiratory distress caused by kidney issues.”* (P06).

The interviewee likened the interdepartmental passing of responsibility to a traditional Chinese martial art, “*Tai Chi.”* (P09). In this situation, departments push responsibilities back and forth, avoiding direct confrontation or resolution.

#### Elevated levels of liability risk

Clinical practice guidelines may have limitations in their applicability to local contexts [46] and sometimes lead to overmedicalization [47]. Interviewees expressed that doctors should not mindlessly adhere to the treatment or examination protocols outlined in clinical practice guidelines, advocating for individualized care tailored to specific circumstances; however, if healthcare professionals have not attached to the guidelines and execute the most comprehensive treatment and examination protocols, they may face responsibility and risks, potentially leading to medical disputes that feel just like “walking on eggshells.“(P07, P08, P09):

*“We basically treat every patient as a potential medical dispute. For instance, if a patient comes in with a headache, based on our many years of experience, we might think it’s just a headache caused by a mild upper respiratory infection. However, we always recommend that the patient undergo additional head CT. If they are unwilling, we have them sign a waiver. We inform the patient or their family about many potential issues we could discover, letting them make the decision and bear the risk themselves.”*(P07).

High levels of liability risk may potentially restrict doctors’ capacity to offer personalized treatment strategies:

*“(In the face of potential medical disputes.) Our communication skills and treatment decisions may change, leading our processes to lean more toward textbook-style treatment. Textbooks and guidelines are often considered guiding principles, but therapy should be flexible. Sometimes, this rigid adherence to textbooks can hinder doctors’ abilities, ultimately to the detriment of patients. However, from a medical-legal perspective, textbooks take precedence over guidelines. Suppose treatment doesn’t follow the textbooks, procedures, and expert consensus. When problems arise, it could ultimately lead to accountability.”* (P06).

### Strategies for addressing moral distress

While moral distress manifests in various ways, each interviewee has a unique perspective on how to mitigate it. The measures most frequently mentioned by the interviewees include enhancing the dissemination of emergency medical knowledge to the general public, refining the social and economic support systems, strengthening multidisciplinary collaboration, and improving doctors’ proficiency in doctor-patient communication are among the measures most frequently endorsed by interviewees.

#### Enhancing the dissemination of emergency medical knowledge to the general public

Enhancing public consciousness regarding emergency medicine will result in improved doctor-patient relationships and more streamlined medical visits:

The senior Surgeon with extensive experience in the ED stated: *“Firstly, educating the public about medical knowledge is beneficial as it enhances their understanding of various illnesses and also promotes their health. Secondly, it helps to improve mutual trust and rapport between doctors and patients, thus fostering better doctor-patient relationships.”*(P02).

The young Surgeon with six months of experience suggested: *“We could conduct more community outreach and educational campaigns to help everyone understand which conditions require a visit to the ED and which don’t. Also, informing people about which illnesses necessitate seeing a specialist is essential. Additionally, organizing triage areas in different zones would be beneficial. This approach aids patients in understanding their situations better, reducing their psychological burden, and expediting their access to care.”*(P03).

#### Refining the social and economic support systems

Some interviewees (P01, P02, P07) suggested that financial assistance for treatment at all levels could be enhanced to assist patients during trying circumstances:

The internal medicine physician with over 10 years of experience mentioned: *“Patients who are critically ill indeed hope for an increase in their reimbursement rates and the removal of some insurance restrictions. When they pay less out of pocket, family members may be less likely to give up easily.”* (P07).

The Surgeon with 5 years of experience suggested: *“Referring to some foreign assistance funds could be beneficial. For patients or their families, having access to these channels allows them to apply for funds that, if their circumstances align, could alleviate some of their medical expenses. This also reduces concerns among healthcare providers about their financial burden (P02).”*

#### Strengthening multidisciplinary collaboration

Most interviewees believe that through coordination with various medical institutions and departments, improving referral and admission plans, and optimizing bed utilization, emergency patients can receive more detailed examinations and treatments in corresponding specialties in a timely manner:

The senior internal medicine physician with 15 years of emergency experience offered valuable insights: *“Making stipulations such as mandating our inpatient department to reserve some beds for emergencies daily or implementing real-time monitoring of available beds information, ensuring transparency in information dissemination.”* (P09).

*“For the inpatient department, perhaps expediting turnover could be possible. Once patients stabilize after managing severe illnesses like post-surgery cases, they could be transferred to other healthcare facilities, such as community or secondary hospitals. Additionally, some departments operate at full capacity with no available beds daily, while others are not as occupied. After extensive discussions, our aspiration is to achieve a ‘hospital with a single bed’ approach, meaning patients with any ailment can be admitted as long as there’s a bed available in any department and specialized doctors can attend to them.”* (P05) The internal medicine physician with a lifelong career in emergency medicine is suggested.

#### Improving doctors’ communication skills

When addressing communication challenges between doctors and patients, most interviewees recognized that these issues are not solely the patients’ fault; doctors also need to empathize with patients and consider their perspectives (P03, P04, P05). They stress the significance of improving communication skills and fostering positive patient relationships:

The young Surgeon believes: *“It’s important for doctors to communicate in simpler terms to help patients understand their condition, and there’s a need to emphasize the significance of thorough examinations and the consequences of incomplete ones. It’s mainly about gradual improvement through step-by-step communication to help patients accept these aspects.”* (P04).

The senior internal medicine physician added: *“I believe our healthcare professionals, especially doctors, could benefit from training to enhance their communication skills. Drawing from their own clinical experiences and presenting real-life examples can significantly improve their ability to communicate effectively. After acquiring such experiences, they can effectively persuade family members and gain their trust.”* (P05).

## Discussion

The study findings indicate that a lack of healthcare resources frequently contributes to moral distress among *ED* doctors. This aligns with the conclusions drawn by scholars from Pakistan and Stockholm [28, 31]. The majority of participants expressed the belief that the scarcity of healthcare resources primarily stems from the insufficient capacity of the workforce to adequately cater to the needs of diverse emergency patients while maintaining high standards of care. Due to the rapid progression of emergency conditions, ED doctors often face significant challenges in making precise diagnoses within short timeframes. In addition, it has become a more substantial challenge due to the inherent complexity of illnesses and a shortage of personnel in ED. Additionally, non-emergency patients occupying ED resources impede timely and quality care for those requiring emergency treatment and escalate the workload for ED doctors, leading to increased job stress. The increasing workload on ED doctors leads to professional burnout, resulting in healthcare staff attrition [48]. This exacerbates the imbalance between emergency medical resources and demand, further increasing the challenge of meeting patient needs effectively.

Almost all interviewees mentioned that communication barriers between doctors and patients are their primary moral distress. They believe that poor communication between healthcare providers and patients can impact treatment outcomes and the quality of care, a viewpoint supported by other qualitative studies [49]. Patients rushed to the ED often present with sudden, urgent, and critical conditions. Their families are unprepared for the severity of the situation, leading to anxiety and an inability to discuss treatment plans with doctors calmly. Additionally, negative media coverage contributes to a negative societal atmosphere, undermining trust between doctors and patients and making effective communication challenging from the outset. A study conducted in China in 2018 also demonstrated that negative news reports had a detrimental effect on perceptions of the doctor-patient relationship [50]. Ultimately, this impacts patients’ and families’ treatment decisions, leading to treatment delays.

Research indicates that in China, Confucian culture tends to regard the family as the fundamental unit of society, with significant decisions regarding individual welfare often made collectively by family members [51]. This study’s findings reveal that, driven by familial emotional needs, some terminally ill patients without prospects of recovery are urged to continue resuscitation efforts. Consistent with other research, Chinese doctors often agree with the requests of family members [52, 53]. In cases where patients are unconscious, determining whether non-essential resuscitation aligns with the patient’s wishes becomes challenging. Apart from emotional factors, some relatives may push for prolonged resuscitation due to financial interests, resulting in inhuman treatment for the patient and the waste of valuable medical resources. In that situation, interviewees were often confused about “what role did I play?”

However, most doctors express that many patients with treatment prospects struggle to maintain optimal care due to some patients’ economic constraints. This perspective aligns with the viewpoints of Pakistani scholars [28]. The interviewed doctors work in a hospital located in southwestern China, where many patients hail from rural or remote areas with lower incomes. Even when the disease itself offers treatment prospects, patients often find it challenging to afford superior yet expensive treatment options. Additionally, doctors emphasize insufficient interdisciplinary collaboration, which partly affects the efficiency of patient referrals after triage in the ED. The inadequacy of interdisciplinary collaboration may stem from unclear treatment roles and practice scopes [54] and the additional workload it brings [55]. Consequently, patients struggle to receive timely intervention from other specialists, while ED doctors find it challenging to provide thorough and specialized treatment. Another aspect contributing to moral distress for ED doctors is the high level of responsibility risk due to conflicts between clinical realities and treatment guidelines. Doctors note that although existing guidelines may apply to most patients, individualized treatment plans often suit patients’ specific clinical circumstances better. However, deviating from current guidelines in treatment decisions places them at risk, akin to “walking on eggshells.”

Responding to the aforementioned moral distress, the interviewees proposed the need to enhance public awareness of emergency medicine. This could be achieved through the widespread dissemination of ED and medical knowledge across various levels. For instance, governments can educate the public on when to seek care at EDs, thereby reducing unnecessary visits [56]. As stated by the WHO, health education is integral to promoting health on both individual and community levels [57]. This could contribute to improved understanding and trust between healthcare providers and patients, fostering better communication. At the societal level, further enhancing the healthcare insurance system by increasing reimbursement rates or establishing relief funds to provide ongoing financial support for eligible patients could alleviate economic burdens, enabling continued treatment. A 2021 study on Chinese healthcare insurance suggests maximizing financial assistance for vulnerable insured individuals [58], aiding patients in overcoming hardships. Internally, strengthening interdisciplinary collaboration among different departments, refining referral protocols, and sharing bed availability information can ensure timely treatment for emergency patients. Lastly, enhancing communication systems through collaborative efforts within healthcare teams is essential [59]. Additionally, providing more communication skills training for ED doctors [60], such as patient-centered communication courses [61], will foster more humanistic care in emergency care settings.

### Limitation

This study has certain limitations. China exhibits spatial heterogeneity in medical technology, economy, culture, and geographical environment [13]. The research is single-center research conducted in an underdeveloped area, and it is necessary to conduct multicenter studies in more developed cities to enhance generalizability. The study explores moral distress from the perspective of ED doctors, and future research could explore this distress from the viewpoints of patients and other disciplines or departments.

## Conclusion

This study provides insight into alleviating moral distress faced by ED doctors. Doctors working in EDs often encounter moral distress. This research indicates the disparity between limited medical resources and the demand for high-quality treatment, ineffective communication between healthcare providers and patients, rescuing patients without therapeutic significance, and challenges in sustaining or implementing optimal treatment measures faced by Chinese ED doctors. Clinical management and policymakers can alleviate this distress by enhancing the dissemination of emergency medical knowledge to the public, refining social and economic support, strengthening multidisciplinary collaboration, and improving doctor-patient communication skills.

### Electronic supplementary material

Below is the link to the electronic supplementary material.

Supplementary Material 1

Supplementary Material 2

## Data Availability

The data generated and analyzed during the current study are available from the corresponding author upon reasonable request.

## Abbreviations

ED: Emergency department
