# Supplementary material for: “I feel like I’m walking on eggshells”: a qualitative study of moral distress among Chinese emergency doctors
Source: BMC Med Ethics. 2024 Jun 20;25:72. doi: 10.1186/s12910-024-01074-4 (PMC11188161; doi:10.1186/s12910-024-01074-4)
Supplement: Supplementary file 1 — Supplementary Material 1 [file 12910_2024_1074_MOESM1_ESM.docx]

**Additional file 1**

**IN-DEPTH INTERVIEW GUIDE**

**Title: “ I feel like I'm walking on eggshells ”: a qualitative study of moral distress among Chinese emergency doctors.**

***Introduction:*** You have been identified as someone who could potentially participate in this study because you are an emergency department(ED) doctor, so you know and have experience relevant to this topic of discussion.

**Background information**

I'm going to ask you a few questions about your background. This information helps us learn about our interviewees (your identity will remain confidential).

|  | Sex (observe) | 1. Male 2. Female 3. Other |
| --- | --- | --- |
|  | Position in ED | 1. Physician  2. Surgeon |
|  | Highest level of education attained | 1. Bachelors 2. Masters 3. MD   Other(specify) ……………………………………………………………………… |
|  | Duration of work in ED | ……………………………………………………………………… |
|  | Titles for Medical Doctors | 1. Junior doctors  2. Intermediate doctors  3. Senior doctors |
|  | How old are you? | ………………… Years  Prefer not to say |

**Interpretation of nouns**

I'm glad you could participate in our research. Before we begin our interview, let me introduce you to the definition of moral distress.

In specific circumstances, individuals face conflicting moral principles or values, leading to moral distress where clear moral choices cannot be made.

• Moral distress typically involves:

o Conflicting moral principles: Individuals confront two or more mutually contradictory moral guidelines, such as honesty versus protecting others' feelings, fairness versus personal interests, etc.

o Dilemma of consequences: Each choice results in different consequences, all of which hold moral significance and value.

o Lack of clear guidance: In moral distress, there are no explicit rules or guidelines for individuals to reference, necessitating personal deliberation and decision-making.

After reading this, you can associate circumstances you have personally encountered, and when you are ready, we can begin our talk.

**Discussion Questions**

**Main questions**

1. Could you provide examples of moral distress you commonly encounter in the ED?

Probing question:

Could you describe these events specifically?

2. What is the frequency of experiencing such events?

Probing question:

1. What kind of emotions do they evoke in you?

2. How do you think these incidents have affected your work and life?

3. What regulations do you refer to when facing these issues?

Probing question:

How do you view these related regulations?

4. What do you believe are the causes of moral distress?

5. In your opinion, what aspects can resolve or improve moral distress in the workplace?

**Addition question**

6. Is there anything else you'd like to add or say concerning the subject of this study?

Your participation in our interview was incredibly beneficial to our research—many thanks in advance.
