# Supplementary material for: “I feel like I’m walking on eggshells”: a qualitative study of moral distress among Chinese emergency doctors
Source: BMC Med Ethics. 2024 Jun 20;25:72. doi: 10.1186/s12910-024-01074-4 (PMC11188161; doi:10.1186/s12910-024-01074-4)
Supplement: Supplementary file 2 — Supplementary Material 2 [file 12910_2024_1074_MOESM2_ESM.docx]

**Additional file 2**

**Consolidated criteria for reporting qualitative studies (COREQ): 32-item checklist (Tong et al. 2007)**

| **No.** | **Item** | **Description** | | **Section #** |
| --- | --- | --- | --- | --- |
| **Domain 1: Research team and reflexivity** | | | | |
| Personal characteristics | | | | |
| 1. | Interviewer/facilitator | *Which author/s conducted the interview or*  *focus group?* | | On page 9; data collection |
| 2. | Credentials | *What were the researcher's credentials?* | | On page 1, authors and affiliations |
| 3. | Occupation | *What was their occupation at the time of the study?* | | On page 1, authors and affiliations |
| 4. | Gender | *Was the researcher male or female*? Both females | | JL, QS, JS and YL are males, FD is female. |
| 5. | Experience and  training | *What experience or training did the researcher have?* | | On page 6, Study Design. |
| Relationship with participants | | | | |
| 6. | Relationship  established | *Was a relationship established prior to study commencement?* | | No |
| 7. | Participant knowledge of the interviewer | *What did the participants know about the researcher? E.g. Personal goals, reasons for doing the research* | | On page 9, data collection. |
| 8. | Interviewer characteristics | *What characteristics were reported about the interviewer/facilitator? E.g. Bias, assumptions, reasons and interests in the research topic* | | / |
| **Domain 2: Study design** | | | | |
| Theoretical framework | | | | |
| 9. | Methodological orientation and theory | *What methodological orientation was stated to underpin the study? E.g. grounded theory, discourse analysis, ethnography, phenomenology, content analysis* | | On page 6, Study design; page 10, analysis. |
| Participant selection | | | | |
| 10. | Sampling | *How were participants selected? E.g. purposive, convenience, consecutive, snowball* | | On page 8 participants. |
| 11. | Method of approach | *How were participants approached? E.g. face- to-face, telephone, mail, email*  Email and, in a second step, telephone calls | | On page 9, data collection. |
| 12. | Sample size | *How many participants were in the study*?  103 ( 41 males and 62 females) | | See Table 1. |
| 13. | Non-participation | *How many people refused to participate or*  *dropped out? What were the reasons for this?* | | None. |
| Setting | | | | |
| 14. | Setting of data  collection | *Where was the data collected? E.g. home, clinic, workplace* | | On page 9, data collection. |
| 15. | Presence of non-  participants | *Was anyone else present besides the*  *participants and researchers?* | | No. |
| 16. | Description of sample | *What are the important characteristics of the sample? E.g. demographic data, date.* See Table n.1 | | See Table 1 |
| Data collection | | | | |
| 17. | Interview guide | | *Were questions, prompts, guides provided by the authors?* Yes  *Was it pilot tested?* Yes. | On page 9, data collection. |
| 18. | Repeat interviews | | *Were repeat interviews carried out? If yes, how many? N.A.* | On page 8, participants |
| 19. | Audio/visual recording | | *Did the research use audio or* *visual recording to collect the data?*  Yes, audio recorded. | On page 9, data collection. |
| 20. | Field notes | | *Were field notes made during and/or after the interview or focus group?* | On page 9, data collection. |
| 21. | Duration | | *What was the duration of the interviews or*  *focus group?* | On page 9, data collection. |
| 22. | Data saturation | | *Was data saturation discussed?* | On page 8, participants |
| 23. | Transcripts returned | | *Were transcripts returned to participants for*  *comment and/or correction*? No | On page 10, analysis. |
| **Domain 3: analysis and findings** | | | | |
| Data analysis | | | | |
| 24. | Number of data  coders | | *How many data coders coded the data?* | Two data coders and 2 reviewers. |
| 25. | Description of the  coding tree | | *Did authors provide a description of the coding tree?* | Yes. |
| 26. | Derivation of themes | | *Were themes identified in advance or derived* *from the data?* | From the data. |
| 27. | Software | | *What software, if applicable, was used to*  *manage the data?* | On page 10, analysis; Nvivo 14 software. |
| 28. | Participant checking | | *Did participants provide feedback on the findings?* | Yes. On page 10, analysis. |
| Reporting | | | | |
| 29. | Quotations presented | | *Were participant quotations presented to illustrate the themes / findings?* Yes. | On page 11-27, findings |
| 30. | Data and findings  consistent | | *Was there consistency between the data presented and the findings?* Yes | On page 11-27, findings |
| 31. | Clarity of major  themes | | *Were major themes clearly presented in the findings?* Yes | On page 11-27, findings |
| 32. | Clarity of minor  themes | | *Is there a description of diverse cases or discussion of minor themes*? Yes | On page 11-27, findings |

Allison Tong et All. Consolidated criteria for reporting qualitative research (COREQ): a 32-item checklist for interviews and focus groups, *International Journal for Quality in Health Care*, Volume 19, Issue 6, December 2007, Pages 349–357, <https://doi.org/10.1093/intqhc/mzm042>
